# An investigation of per- and polyfluoroalkyl concentrations in dogs and cats with endocrine disorders

**DOI:** 10.3389/fvets.2026.1904721

**Published:** 2026-08-24

**Authors:** Anisa Bardhi, Elena Serrantoni, David Inauen, Francesca Del Baldo, Sara Galac, Ronette Gehring, Federico Fracassi, Andrea Barbarossa

**Affiliations:** 1 Department of Veterinary Medical Sciences, University of Bologna, Bologna, Italy; 2 Department of Population Health Sciences, Division of Veterinary Pharmacology and Pharmacy, Faculty of Veterinary Medicine, Utrecht, Netherlands; 3 Department of Clinical Sciences, Faculty of Veterinary Medicine, Utrecht University, Utrecht, Netherlands

**Keywords:** biological fluids, companion animals, emerging contaminants, internal medicine, One Health

## Abstract

**Introduction:**

Per- and polyfluoroalkyl substances (PFAS) are persistent environmental contaminants with endocrine-disrupting and potential carcinogenic effects in humans. Data on PFAS exposure in companion animals are limited. This study quantified PFAS concentrations in dog and cat serum and investigated associations with endocrine disorders.

**Methods:**

This retrospective study included 179 dogs from the Universities of Bologna and Utrecht, and 84 cats from the University of Bologna, sampled between 2021 and 2025. The population comprised 23 healthy dogs, 35 healthy cats, and animals with endocrine disorders, including 49 cats with hyperthyroidism, 64 dogs with pituitary-dependent hypercortisolism, 16 with primary hyperparathyroidism, and 85 with adrenal tumors. Eleven PFAS were measured by liquid chromatography–tandem mass spectrometry (LC–MS/MS). Between-group differences were evaluated using nonparametric tests, associations with disease status using age-adjusted generalized linear models, and correlations with age and body condition score using Spearman’s rank correlation.

**Results:**

Eight PFAS were detected in dogs and nine in cats. The most frequently detected compounds were perfluorooctanesulfonic acid (PFOS, 99.5%), perfluorohexanesulfonic acid (PFHxS, 69.0%), and perfluorooctanoic acid (PFOA, 68.5%) in dogs, and PFOS (99.0%) and PFOA (96.0%) in cats. Dogs from Utrecht had higher PFOS concentrations than dogs from Bologna. Unadjusted between-group differences varied by species, with higher PFAS concentrations in healthy cats but higher concentrations of some PFAS in certain canine diseased groups. After adjustment for age, no PFAS was independently associated with disease status in either species. A weak negative correlation between ∑PFAS and age was observed in dogs (*r* = −0.206, *p* = 0.005) and cats (*r* = −0.234, *p* = 0.032), and diseased animals were older than healthy controls, suggesting that age contributed to the observed between-group differences. No significant associations were found with body condition score.

**Discussion and conclusions:**

PFAS exposure was widespread in both species, with higher PFOS concentrations in dogs from the Dutch cohort. The inverse association between PFAS concentrations and age, together with the older age of diseased animals, suggests that age substantially contributed to the observed between-group differences. Further longitudinal studies, including minimally exposed control populations, are warranted to clarify the health implications of PFAS exposure in companion animals.

## 1 Introduction

Per- and polyfluoroalkyl substances (PFAS), commonly known as *“forever chemicals,”* are a large class of synthetic compounds extensively used in industrial applications and consumer products. They are characterized by strong carbon–fluorine bonds, which confer exceptional resistance to thermal, chemical, and biological degradation (1). As a result, PFAS persist in the environment, bioaccumulate in living organisms, and have been linked to potential diverse adverse health effects (2–6). Regulatory agencies, including the European Food Safety Authority (EFSA) and the U.S. Environmental Protection Agency (EPA), have established monitoring programs to assess PFAS exposure, evaluate health risks, and guide regulatory actions (7–9).

Among PFAS, perfluorooctanesulfonic acid (PFOS) and perfluorooctanoic acid (PFOA) have received particular attention due to their toxicological profiles. The International Agency for Research on Cancer (IARC) classified PFOA as carcinogenic to humans (Group 1) and PFOS as possibly carcinogenic to humans (Group 2B) (10, 11). Epidemiological studies have reported associations between PFAS exposure and several human health outcomes, including thyroid cancer and other neoplastic conditions, although the strength and causality of these relationships remain under investigation (12).

PFAS are considered endocrine-disrupting chemicals (EDCs) due to their ability to interfere with hormonal regulation through multiple mechanisms. Owing to their amphiphilic, fatty-acid-like structure, PFAS bind with high affinity to plasma transport proteins and nuclear receptors, most notably the peroxisome proliferator-activated receptors (PPARα and PPARγ), which regulate lipid metabolism, steroidogenesis, and cellular differentiation (13). In addition, PPARα and PPARγ are expressed in the adrenal cortex, where they participate in the regulation of enzymes involved in glucocorticoid and mineralocorticoid biosynthesis, altering the secretion of cortisol precursors, aldosterone and other adrenal steroids (13, 14). PFAS may also compete with thyroxine (T4) for binding sites on transthyretin (TTR), potentially altering thyroid hormone transport and affecting the hypothalamic–pituitary–thyroid (HPT) axis (15–17). Consistent with these mechanisms, PFAS exposure has been associated with altered glucocorticoid and progestogen concentrations in newborns, and endocrine-disrupting chemicals more broadly are recognized to target the entire adrenocortical steroidogenic cascade, from the ACTH receptor and steroidogenic acute regulatory protein to the mitochondrial cytochrome P450 enzymes (18).

Although the precise mechanisms remain incompletely understood, these findings provide a biological rationale for investigating the relationship between PFAS exposure and a broader spectrum of endocrine disorders. Humans and animals may be exposed to PFAS through several pathways, with dietary intake considered the primary route, particularly for persistent long-chain compounds such as PFOS and PFOA (19–22). Other relevant routes include ingestion of contaminated household dust, inhalation of indoor air, and dermal contact with PFAS-containing materials (7, 23–25). Breast milk represents another important elimination and transfer pathway, contributing to the passage of these compounds from mothers to offspring (25–27). In domestic environments, PFAS may originate from numerous sources, including fluorinated textiles, stain- and water-resistant treatments, food packaging materials, household dust, and contaminated food and drinking water. Indoor sources may account for a substantial proportion of total PFAS exposure, with some studies estimating household-related sources to contribute up to 50% of overall intake (23, 28–31). Although PFAS-related health effects have been extensively investigated in humans, data on their occurrence, accumulation, and potential biological effects in companion animals remain limited.

Compared with humans, information regarding PFAS concentrations, accumulation, and biological effects in companion animals remains limited. Because dogs and cats share indoor environments with humans, they are likely to experience similar PFAS exposure patterns and may serve as valuable sentinels of environmental contamination within a One Health framework (32–34). Companion animals may also be exposed through grooming behavior and the ingestion of household dust (35). Associations between PFAS concentrations in household dust and serum concentrations of specific compounds, such as PFOA and PFUnDA, have been reported in companion animals, suggesting that indoor contamination represents a relevant exposure pathway. Diet and contaminated water may also contribute to PFAS body burdens, particularly for PFOS and PFHxS (35–37).

Endocrine disorders are among the most frequently diagnosed chronic diseases in small animal practice. Feline hyperthyroidism (FHT) is the most common endocrine disorder in older cats, affecting approximately 10–12% of cats over 10 years of age (38). In dogs, naturally occurring hypercortisolism is one of the most frequently recognized endocrine diseases, with an estimated prevalence of approximately 0.2–0.3% in primary-care populations (39, 40). Other disorders, including primary hyperparathyroidism and functional adrenal tumors, are less common but clinically relevant due to their systemic effects, diagnostic complexity, and therapeutic implications.

Although previous studies have suggested a possible association between PFAS exposure and thyroid dysfunction in animals, the potential involvement of these contaminants in other endocrine diseases remains largely unexplored. Chronic exposure to PFAS has already been associated with conditions such as feline hyperthyroidism (35, 37). Moreover, the documented effects of PFAS on thyroid hormone transport, nuclear receptor signaling, and steroidogenesis provide a biological rationale for investigating endocrine disorders affecting multiple organs, including the thyroid, adrenal glands, and parathyroid glands. This may provide further insight into the potential role of environmental contaminants in endocrine dysfunction and tumorigenesis in companion animals.

In this context, the present study aimed to quantify PFAS concentrations in canine and feline serum samples and to investigate potential associations between PFAS exposure and a broad spectrum of endocrine disorders, including feline hyperthyroidism (FHT), primary hyperparathyroidism (PHPT), pituitary-dependent and adrenal-dependent hypercortisolism (PDH and ADH), non-secretory adrenal tumors (NST), pheochromocytoma (PCC), and mixed forms of hypercortisolism (mHAC). By doing so, the study sought to provide insight into environmental exposure patterns and their potential pathophysiological relevance in companion animals.

## 2 Materials and methods

### 2.1 Study population

This retrospective study included dogs and cats diagnosed with endocrine disorders. Medical records from February 2021 to March 2025 were reviewed from the Veterinary Teaching Hospital of the University of Bologna and the Department of Clinical Sciences of the Faculty of Veterinary Medicine of Utrecht University. Data collected included signalment, clinical history, diagnostic evaluations.

Demographic and clinical data were retrospectively collected from the medical records. The recorded variables included breed, age, sex, reproductive status, housing conditions, diet, diagnosis, comorbidities, and Body Condition Score (BCS). BCS was assessed by trained clinicians using a 9-point scale. Information on endocrine disorders and concurrent diseases was obtained from the diagnoses documented in the medical records.

The study population was divided into two groups: animals with endocrine disorders and healthy controls. The healthy control group consisted of 35 cats and 23 dogs. Animals were considered healthy if they had no history or clinical evidence of acute or chronic disease. In particular, the absence of neoplastic disease and clinically relevant metabolic disorders, including diabetes mellitus and chronic kidney disease, was confirmed. All control animals were examined at the Veterinary Teaching Hospital of the University of Bologna and had not received any medications other than routine vaccinations and ectoparasite/endoparasite prophylaxis. Health status was confirmed based on the medical records, including physical examination findings, complete blood count, serum biochemistry profile, and urinalysis. The endocrine disorder group consisted of privately owned dogs and cats with a confirmed diagnosis of one or more of the following endocrine disorders: PHPT, PDH, ADH, NST, PCC, or mHaC. A total of 126 diagnoses from the University of Bologna and 53 from the University of Utrecht were included (median age: 10 years; range: 2–18 years). The distribution of the study population among the different endocrine disorder categories and sampling centers is reported in Table 1.

**Table 1:** Distribution of dogs included in the study according to geographical origin and health status/endocrine disorder category.

Country	H	PHPT	PDH	AT	PCC	ADH	NST	mHaC	Total
Italy	23	16	51	45	12	20	7	6	135
The Netherlands	0	0	13	40	18	10	12	0	53
Total	23	16	64	85	30	30	19	6	179

Data are reported as number of diagnoses, as some dogs had more than one disorder. The study population included healthy dogs (H) and dogs affected by primary hyperparathyroidism (PHPT), pituitary-dependent hyperadrenocorticism (PDH), adrenal tumors (AT), pheochromocytoma (PCC), adrenal-dependent hyperadrenocorticism (ADH), non-secretory adrenal tumors (NST), and mixed hyperadrenocorticism (mHaC). Adrenal tumors (AT) represent an overall category including PCC, ADH, NST, and mixed hyperadrenocorticism (mHaC); therefore, these categories are overlapping and should not be summed.

Privately-owned cats from the Veterinary Teaching Hospital of the University of Bologna were included if they were ≥8 years old (range: 8–22 years). A group of cats (*n* = 49) affected by FHT was selected using diagnostic procedures consistent with those described in the literature (41, 42).

Diagnoses were established by veterinary endocrinologists at the Veterinary Endocrinology Units of the University of Bologna and Utrecht University using standard diagnostic criteria (43).

### 2.2 Diagnosis of endocrine disorders

The diagnosis of primary hyperparathyroidism (PHPT) was made based on a combination of various laboratory findings, such as the presence of total (and ionized) hypercalcemia alongside low (or normal) phosphorus levels, and endocrine tests showing elevated parathyroid hormone (PTH) levels (55).

Dogs were diagnosed with hypercortisolism (HC) based on compatible clinical signs and the results of endocrine testing. To be included, dogs had to present at least two/three (38) clinical signs consistent with HC (polyuria/polydipsia, polyphagia, alopecia, thin skin, and/or excessive panting) together with at least one abnormal endocrine screening test, including a low-dose dexamethasone suppression test (LDDST), an ACTH stimulation test (ACTHst), or an increased urine cortisol-to-creatinine ratio (UCCR). The differentiation between pituitary-dependent hypercortisolism (PDH) and adrenal-dependent hypercortisolism (ADH) was based on a combination of abdominal ultrasonography, LDDST results, and endogenous ACTH concentrations. In dogs with ADH, the diagnosis was confirmed by imaging findings consistent with an adrenal tumor and, when available, histopathological examination of adrenal tissue.

Dogs were diagnosed with pheochromocytoma (PCC) based on either histopathological confirmation or a combination of compatible clinical findings, diagnostic imaging, and biochemical testing. Histopathological diagnosis was established by hematoxylin and eosin (H&E) staining and, when necessary, confirmed by immunohistochemistry. In dogs without histopathological confirmation, a clinical diagnosis of PCC was based on compatible clinical and clinicopathological findings, the presence of an adrenal mass identified by abdominal ultrasonography or cross-sectional imaging, and increased plasma free or urinary fractionated normetanephrine concentrations consistent with PCC (44, 45).

Dogs were classified as having non-secreting adrenal tumors (NST) in the absence of clinical signs of endocrine disease, with negative endocrine testing, including a normal low-dose dexamethasone suppression test (LDDST), normal endogenous ACTH (eACTH) concentrations, and normal plasma free or urinary fractionated normetanephrine concentrations. When available, the diagnosis was confirmed by histopathological examination demonstrating an adrenocortical tumor.

The diagnosis of feline hyperthyroidism (FHT) was based on a combination of the patient’s medical history and compatible clinical signs, such as weight loss, polyphagia, vomiting and behavioral changes (46). The diagnosis was confirmed by the detection of elevated T4 (39, 40, 42).

### 2.3 Sample collection

Serum samples from dogs and cats were obtained by venipuncture during specialist endocrinology consultations. Blood was collected from the jugular, cephalic, or saphenous veins and allowed to clot in serum-separator tubes, which were subsequently centrifuged at 3,000 × *g* for 10 min. The serum was then transferred to plastic tubes and stored at −20 °C in the clinical laboratory of the respective veterinary hospital. Samples collected in Utrecht were shipped to Bologna on dry ice and stored at −20 °C until LC–MS/MS analysis.

### 2.4 Sample treatment and LC–MS/MS analysis

Serum samples were extracted following the procedure described by Bardhi et al. (47). Briefly, 200 μL of serum was mixed with 20 μL of internal standard and 20 μL of methanol. Proteins were precipitated with 400 μL of acetonitrile and the mixture was centrifuged at 21,000 × *g* for 15 min at 20 °C. A 450 μL aliquot of the supernatant was transferred, evaporated to dryness under nitrogen at 45 °C, and reconstituted in 250 μL of mobile phase (80:20 water:methanol, *v/v*) prior to analysis by Ultra-High-Performance Liquid Chromatography coupled with Tandem Mass Spectrometry (UHPLC–MS/MS; Waters Corporation, Milford, MA, USA).

The target PFAS analytes included perfluorobutanoic acid (PFBA), perfluorobutanesulfonic acid (PFBS), perfluoropentanoic acid (PFPeA), perfluorohexanoic acid (PFHxA), perfluorohexanesulfonic acid (PFHxS), perfluoroheptanoic acid (PFHpA), perfluorooctanoic acid (PFOA), perfluorooctanesulfonic acid (PFOS), perfluorononanoic acid (PFNA), perfluorodecanoic acid (PFDA), and hexafluoropropylene oxide dimer acid (GenX). Analyses were performed in multiple reaction monitoring (MRM) mode, with two transitions monitored for each analyte, one for quantification and one for confirmation.

The method was fully validated in accordance with European guidelines (48), providing satisfactory results for all evaluated validation parameters. The PFAS calibration curves (range 0.1–100 μg/L for both species) exhibited a coefficient of determination (*R*^2^) ≥ 0.99, demonstrating the method’s linearity across the tested concentration range and over three separate days. The limit of detection (LOD) and the lower limit of quantification (LLOQ) were determined based on the signal-to-noise (S/N) ratio. A S/N ratio of approximately 3:1 was considered acceptable for the estimation of the LOD, whereas a minimum S/N ratio of 10:1 was required for reliable quantification. The LLOQ was further confirmed by evaluating accuracy and precision based on four replicate injections, with acceptable values within ±20% and CV < 20%, respectively. For all 11 PFAS analytes included in the study (Supplementary Table S1), the LLOQ was established at 0.1 μg/L, corresponding to a LOD of 0.033 μg/L.

The intra- and inter-day accuracy and precision were satisfactory for all PFAS analytes evaluated. Accuracy values were within ±15% of the nominal concentrations at all quality control (QC) levels, while precision values, expressed as coefficients of variation (CV%), were consistently below 15%. No carry-over was observed during method validation, as no detectable signal was obtained in blank samples injected immediately after the highest calibration standard.

Potential pre-analytical contamination was evaluated through procedural blanks processed alongside serum samples. Background PFAS contributions from reagents and laboratory materials were assessed and accounted for during data analysis. Overall, the method proved reliable and reproducible for PFAS quantification and fulfilled the predefined validation criteria. Further details on the validation procedure are available in Bardhi et al. (47).

### 2.5 Statistical analysis

Statistical analyses were performed using commercial and open-source software (GraphPad Prism 10, R 4.4.2) and nonparametric tests were used. Data are presented as median and range (minimum and maximum value). Descriptive statistics were generated to characterize the study population. For dogs only, the association between location (Bologna, Utrecht) and PFAS concentrations was tested with a Wilcoxon test for each chemical, and the Holm-Bonferroni method was applied to adjust the -values, accounting for multiple comparisons regarding the different PFAS. Health condition probabilities of dogs (Healthy, AT, PDH, PHPT) regarding PFAS concentrations were modeled with a multinomial generalized linear model (GLM), reducing bias with the Poisson trick (49); for health conditions of cats (Healthy, FHT), a binomial GLM was applied. Independent variables were the concentrations of different PFAS; only PFAS with median concentrations greater or equal 0.05 were included. Age of the individual dogs or cats was added as covariate. The Holm-Bonferroni method was applied to adjust the *p*-values; age was not considered during the multiple comparisons’ correction.

The Mann–Whitney test was used to compare PFAS concentrations between the different pathological groups, considering both individual chemical species and cumulative concentrations (∑PFAS). The same test was applied to assess differences in age between groups.

Correlations between PFAS concentrations and numerical variables, such as age and Body Condition Score (BCS), were assessed using Spearman’s rank correlation. A *p*-value ≤ 0.05 was considered statistically significant for all analyses.

For statistical analyses, detected concentrations below the LLOQ were replaced with LLOQ/2 (0.050 μg/L), whereas non-detected values were assigned a value of zero. These substituted values were used to calculate ΣPFAS concentrations and perform correlation analyses.

## 3 Results

In the canine population from the University of Bologna, 69 were female (56 spayed) and 57 were male (25 castrated).

Forty-two were mixed breeds, and 84 were purebred, representing 40 different breeds. Further descriptive characteristics of the study population are provided in Supplementary Table S2.

Among the dogs enrolled in the study, 23 were healthy, 64 had been previously diagnosed with PDH, 16 with PHPT, and 85 with an adrenal tumor (AT), including 30 with PCC, 30 with ADH, 19 with NST, and 6 with mHAC. Nine dogs were affected by more than one endocrine disorder, classified as “AT+PDH.” One of those dogs was also diagnosed with PHPT; for estimation purposes the condition of that individual was also classified as “AT+PDH.” The median age was 10 years (range, 2–18 years), and the median Body Condition Score (BCS) was 5/9 (range, 3/9–8/9).

Among the cats, 35 were healthy and 49 were hyperthyroid. The median age was 14 years (range, 8–22 years), and the median BCS was 4/9 (range, 1/9–8/9). Forty-two cats were female (39 spayed) and 42 were male (38 castrated). 72 Domestic Shorthair, 3 Chartreux, 3 Birman, 2 Maine Coon, 1 Devon Rex, 1 British Shorthair, 1 Siamese, 1 Siberian. Further descriptive characteristics of the feline study population are provided in Supplementary Table S3.

Median and range concentrations of the 11 PFAS, together with the percentage of quantifiable samples, are presented in Tables 2, 3 for dogs and cats, respectively. Differences between dogs from the Universities of Bologna and Utrecht are presented in Supplementary Table S4.

**Table 2:** Median concentrations, ranges, and percentage of quantifiable samples for 11 per- and polyfluoroalkyl substances measured in dog serum samples.

Compound	Median (range, μg/L)	Detection frequency (%)
PFOS	1.765 (0.050–26.596)	99.5
PFOA	0.158 (0.000–8.050)	68.5
PFDA	0.135 (0.000–8.046)	60.0
PFNA	0.131 (0.000–3.098)	60.0
PFBS	0.050 (0.000–1.211)	34.0
PFHxS	0.430 (0.000–4.873)	69.0
PFHxA	0.000 (0.000–0.000)	0.0
PFHpA	0.000 (0.000–0.102)	0.5
PFPeA	0.000 (0.000–0.050)	0.0
GenX	0.000 (0.000–0.584)	1.0
PFBA	0.000 (0.000–0.000)	0.0
ΣPFAS	2.750 (0.231–29.141)	–

ΣPFAS represents the sum of all detected analyte concentrations. Concentrations are expressed in μg/L.

**Table 3:** Median concentrations, ranges, and percentage of quantifiable samples for 11 per- and polyfluoroalkyl substances measured in cat serum samples.

Compound	Median (range, μg/L)	Detection frequency (%)
PFOS	0.766 (0.050–5.138)	99.0
PFOA	0.523 (0.050–29.366)	96.0
PFDA	0.105 (0.050–12.682)	50.0
PFNA	0.179 (0.050–7.517)	71.0
PFBS	0.000 (0.000–0.648)	1.0
PFHxS	0.050 (0.000–2.536)	48.0
PFHxA	0.000 (0.000–0.000)	0.0
PFHpA	0.131 (0.000–4.563)	64.0
PFPeA	0.050 (0.000–0.353)	9.5
GenX	0.000 (0.000–0.050)	0.0
PFBA	0.000 (0.000–2.491)	6.0
ΣPFAS	2.342 (0.412–52.866)	–

ΣPFAS represents the sum of all detected analyte concentrations. Concentrations are expressed in μg/L.

**Figure 1:**
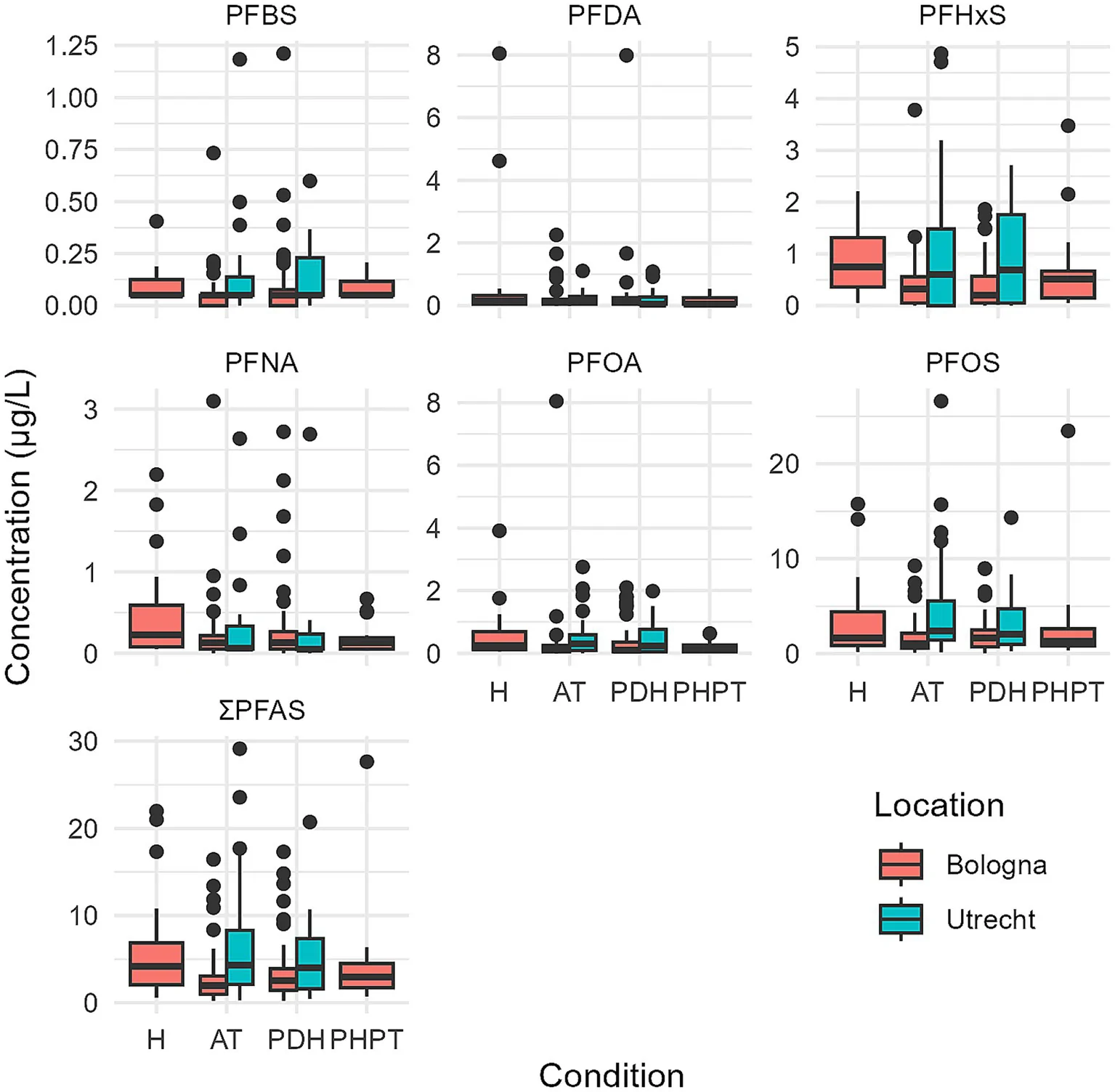
Comparison of per- and polyfluoroalkyl substances concentrations (μg/L) between healthy dogs (H) and those affected by pituitary-dependent hyperadrenocorticism (PDH), primary hyperparathyroidism (PHPT), and adrenal tumors (AT) in the canine population of the Universities of Bologna and Utrecht.

PFOS concentrations in dogs were significantly higher in dogs from the Utrecht University compared to dogs from the University of Bologna, with an adjusted *p*-value of 0.0087 (Figure 1). In unadjusted comparisons, some PFAS concentrations were higher in the PDH and adrenal tumor groups than in healthy dogs. However, in the age-adjusted multinomial model, none of PFBS, PFDA, PFHxS, PFNA, PFOA, or PFOS was independently associated with health status, either before or after correction for multiple comparisons. Dogs with PHPT were significantly older than healthy dogs (*p*-value = 0.001) (Table 4, 5).

**Table 4:** Results of the statistical analysis comparing healthy (H) dogs and those affected by pituitary-dependent hyperadrenocorticism (PDH), primary hyperparathyroidism (PHPT), and adrenal tumors (AT) in the canine population of Bologna.

PFAS	H (*n* = 23)	PDH (*n* = 64)	*p*-value	PHPT (*n* = 16)	p-value	AT (*n* = 85)	*p*-value
PFOS	1.665 (0.165–15.770)	1.665 (0.100–8.965)	0.3363	1.310 (0.3650–23.47)	0.5769	1.065 (0.113–9.265)	0.0829
PFOA	0.242 (0.050–3.912)	0.103 (0.000–2.100)	**0.0243**	0.141 (0.050–0.632)	0.0789	0.138 (0.000–8.050)	**0.0135**
PFDA	0.158 (0.050–8.046)	0.137 (0.050–7.988)	0.6637	0.050 (0.050–0.536)	0.1792	0.120 (0.000–2.256)	0.2252
PFNA	0.227 (0.050–2.196)	0.131 (0.000–2.722)	0.1606	0.131 (0.050–0.668)	0.1814	0.128 (0.000–3.098)	0.0523
PFBS	0.050 (0.050–0.405)	0.050 (0.000–1.211)	0.0671	0.050 (0.050–0.208)	0.7874	0.050 (0.000–0.733)	**0.0074**
PFHxS	0.747 (0.050–2.212)	0.201 (0.000–1.863)	**0.0002**	0.513 (0.050–3.477)	0.0934	0.321 (0.000–3.781)	**0.0005**
ΣPFAS	4.180 (0.574–21.989)	2.723 (0.213–17.309)	0.1607	3.199 (0.715–27.652)	0.2600	1.901 (0.213–29.141)	**0.0120**

Data are reported as median values with minimum–maximum concentration ranges (μg/L), together with the corresponding *p*-values. Significant *p*-values are reported in bold. The number of animals is indicated as *n*.

**Figure 2:**
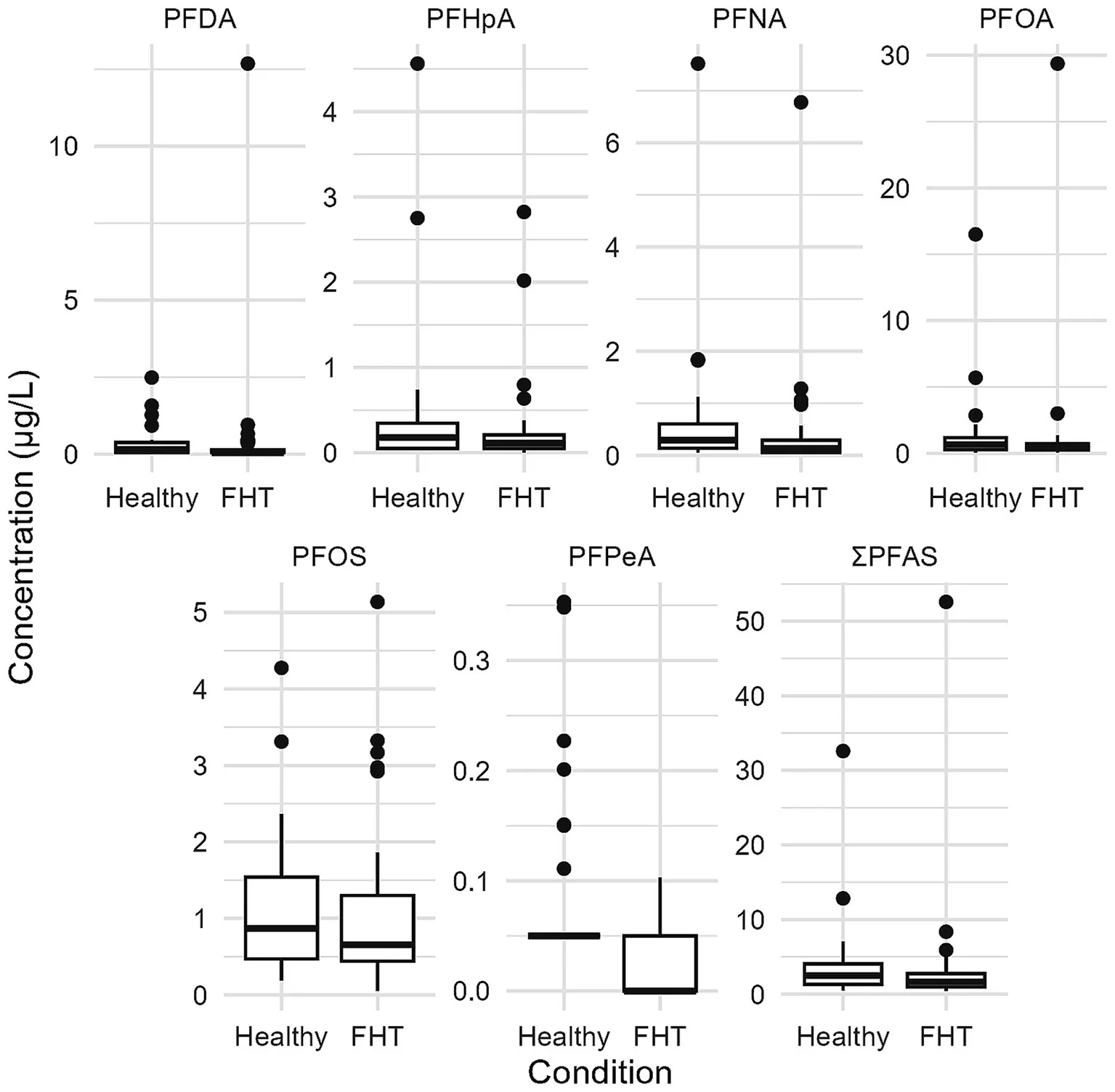
Comparison of serum per- and polyfluoroalkyl substances concentrations (μg/L) between healthy cats and cats with feline hyperthyroidism (FHT).

In unadjusted comparisons, healthy cats generally showed higher concentrations of several PFAS than cats with FHT. In the age-adjusted binomial model, none of PFDA, PFHpA, PFOA, or PFOS was independently associated with health status, either before or after correction for multiple comparisons. PFNA was associated with health status before correction (*p* = 0.046), but this association was no longer significant after adjustment for multiple comparisons. PFPeA was not included in the model because it almost perfectly separated the health-status groups (Figure 2). Healthy cats were significantly younger than cats with FHT (Table 6).

**Table 6:** Results of the statistical analysis comparing healthy cats and those affected feline hyperthyroidism (FHT) in the feline population of Bologna.

	Healthy (*n* = 35)	FHT (*n* = 49)	*p*-value
PFOS	0.867 (0.187–4.275)	0.656 (0.050–5.138)	0.2943
PFOA	0.661 (0.050–16.502)	0.426 (0.050–29.366)	0.1492
PFDA	0.153 (0.050–2.491)	0.050 (0.050–12.682)	**0.0401**
PFNA	0.294 (0.050–7.517)	0.134 (0.050–6.775)	**0.0075**
PFHxS	0.000 (0.000–1.375)	0.127 (0.000–2.536)	0.2293
PFHpA	0.178 (0.050–4.563)	0.113 (0.000–2.823)	0.0885
PFPeA	0.050 (0.050–0.353)	0.000 (0.000–0.103)	**<0.0001**
ΣPFAS	2.784 (0.447–33.600)	1.912 (0.412–52.450)	0.0803

Data are reported as median values with minimum–maximum concentration ranges (μg/L), together with the corresponding *p*-values. Significant *p*-values are reported in bold. The number of animals is indicated as *n*.

**Table 5:** Results of the statistical analysis comparing healthy dogs and dogs affected by pheochromocytoma (PCC), adrenal-dependent hyperadrenocorticism (ADH), and mixed forms of hyperadrenocorticism (NST and mHAC) in the canine population from Bologna.

	Condition	Diagnosis	*p*-value
PFAS	Healthy	PCC (*n* = 30)	
ΣPFAS	4.180 (0.574–21.989)	1.908 (0.414–5.410)	0.0141
		ADH (*n* = 30)	
		2.396 (0.328–13.394)	0.1631
		NST (*n* = 19)	
		2.483 (1.422–16.439)	0.5645
		mHAC (*n* = 6)	
		0.8740 (0.213–1.771)	**0.0027**

Data are reported as median values with minimum–maximum concentration ranges (μg/L), together with the corresponding *p*-values. Significant *p*-values are reported in bold. The number of animals is indicated as *n*.

**Figure 3:**
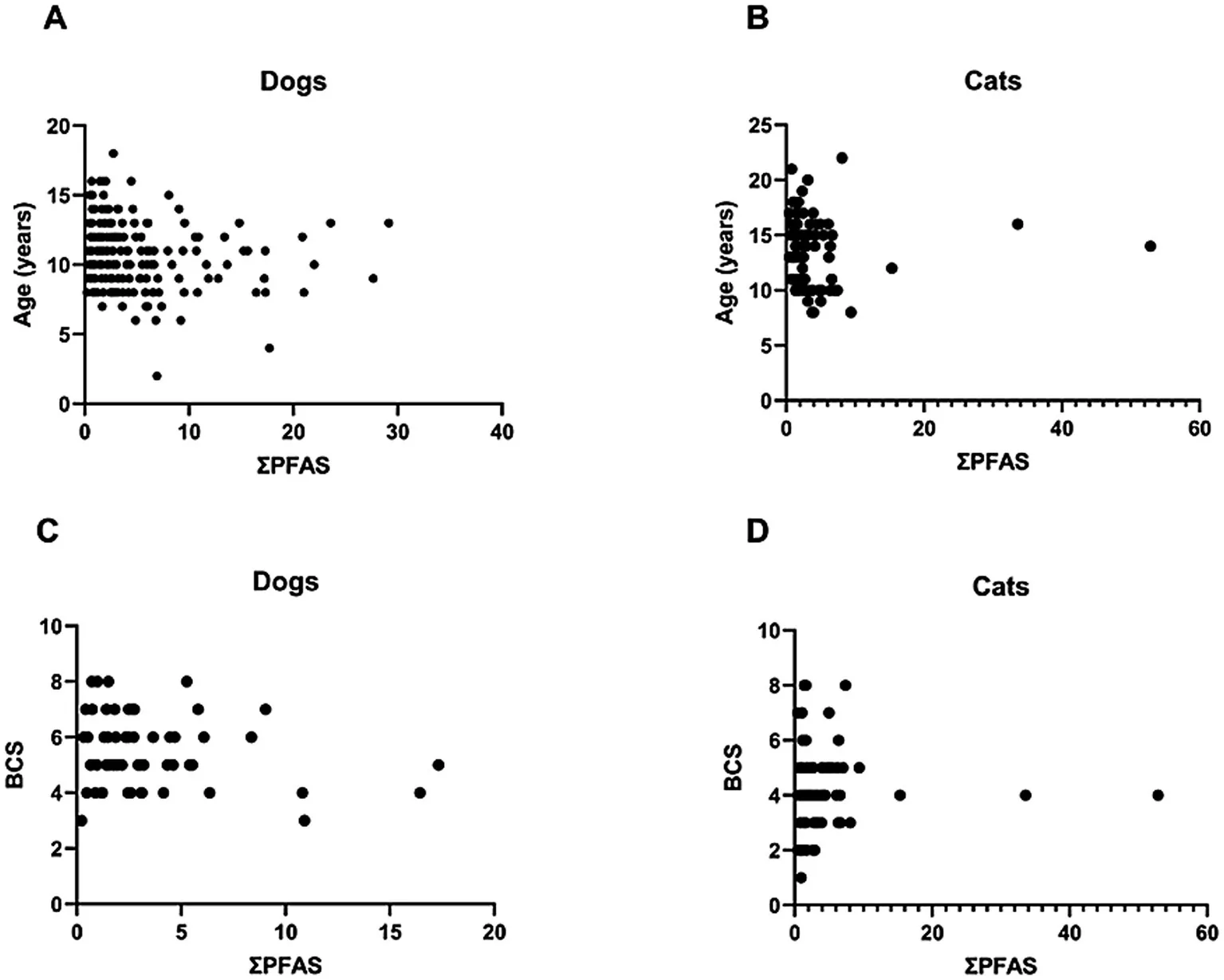
Correlation between age (years), Body Condition Score (BCS), and ∑PFAS concentrations (μg/L) in dogs and cats.

In dogs, a significant negative correlation was observed between ∑PFAS concentrations and age (*r* = −0.2058; *p* = 0.0054) (Figure 3).

In cats, no significant associations were observed between ∑PFAS concentrations and BCS. However, a weak significant negative correlation was found between ∑PFAS concentrations and age (*r* = −0.2337; *p* = 0.0324). Furthermore, both healthy dogs (*p* = 0.0174) and healthy cats (*p* = 0.0066) were significantly younger than their diseased counterparts. Thus, the inverse association between ∑PFAS concentrations and age, together with the older age of diseased animals, suggests that age substantially contributed to the observed between-group differences (Table 6).

## 4 Discussion

This study confirmed the presence of 8 out of 11 PFAS compounds in dog serum and 9 out of 11 in cat serum. The most frequently detected molecules in dogs were PFOS (99.5%), PFHxS (69%), and PFOA (68.5%), which is consistent with existing literature (33). In cats, dominant molecules were PFOS (99%) and PFOA (96%). Some differences were observed between the two species. While the dominant compounds were the same, PFBS was more prevalent in dogs, in which the median ΣPFAS concentration was 2.750 μg/L. In contrast, most perfluoroalkyl carboxylic acids (PFBA, PFPeA, PFHpA, PFOA, and PFNA) were more frequently, or exclusively, detected in cats. This finding aligns with previous studies showing higher PFCA exposure in cats, likely due to fish-based diets (50).

When comparing PFAS concentrations between dogs enrolled at the University of Bologna and Utrecht University, PFOS concentrations were significantly higher in dogs from Utrecht University (adjusted *p*-value = 0.0087). No significant associations between geographical locations and the concentrations of the other PFAS, either before or after *p*-value adjustment. These findings may indicate geographical differences in PFOS exposure patterns; however, further studies involving larger populations and more detailed exposure assessments are needed to clarify the factors underlying these differences and to better understand the potential role of PFAS in canine health.

The unadjusted comparisons differed in direction by species. Healthy cats generally showed higher concentrations of several PFAS than cats with FHT, whereas concentrations of some PFAS were higher in the PDH and adrenal tumor groups than in healthy dogs. However, no PFAS was independently associated with disease status in either species after adjustment for age.

In the feline age-adjusted model, PFDA, PFHpA, PFOA, and PFOS were not significantly associated with health status, either before or after adjustment for multiple comparisons. Although PFNA showed a significant association before correction, this was no longer significant after adjustment for multiple comparisons, suggesting that it may represent a spurious finding. PFPeA was excluded from the GLM due to near complete separation between health-condition groups. Furthermore, the younger age of healthy cats compared with cats affected by FHT should be considered a potential confounding factor when interpreting these results. The results also revealed a weak negative correlation between age and ΣPFAS levels in both dogs and cats. This may be related to the elimination mechanisms of per- and polyfluoroalkyl substances in pets, which are not yet fully understood. It is possible that these substances are eliminated relatively quickly in dogs and cats, a hypothesis supported by the shorter half-lives reported in the literature (34). Existing veterinary literature in dogs shows mixed evidence regarding correlations with age (51), with results varying depending on the chemical species and sex. However, many other studies report no significant associations (48, 50, 51). In cats, our findings contrast with previous reports, which found no correlation between age and PFAS levels (35).

In humans, PFAS levels generally increase with age due to accumulation, although some studies suggest a non-linear trend, such as a J-curve, in which concentrations decrease during early life and rise sharply later (52). In our study, ∑PFAS concentrations were negatively correlated with age in both species, and diseased animals were significantly older than healthy controls. These findings suggest that age substantially influenced the observed between-group differences and do not support either a general conclusion of higher PFAS concentrations in healthy animals or an independent association with endocrine disease status.

No significant associations were found between ΣPFAS concentrations and BCS in dogs, consistent with previous studies (53). In cats, only one study has investigated the association with BCS, showing a positive correlation with PFAS concentrations, in contrast with our findings (54). The lack of observed associations may be due to the analysis being limited to ΣPFAS rather than individual chemical species, as previous studies often analyzed each PFAS compound separately and stratified by sex.

Despite providing valuable insights into PFAS exposure in companion animals, this study has some important limitations. The retrospective design restricted follow-up and complete data collection, resulting in variability in the timing of blood sampling, as samples were obtained either at diagnosis or during medical follow-up. Although follow-up samples were collected from dogs receiving medical treatment, the potential influence of treatment timing on PFAS concentrations cannot be completely excluded. Furthermore, the wide variability in PFAS concentrations among healthy Italian control animals highlights the difficulty of identifying minimally exposed control populations. Another limitation is the lack of systematically collected information on potential determinants of PFAS exposure, including diet, housing conditions, and geographic origin. Owing to the retrospective nature of the study, these variables were unavailable for a substantial proportion of animals, preventing robust analyses and potentially introducing selection bias. The limited age distribution hindered a thorough assessment of age-related associations, while the absence of healthy dogs from Utrecht University constrained regional comparisons.

Future studies should aim for broader geographic sampling within Italy and beyond, and should incorporate well-defined, minimally exposed control populations. Addressing these limitations will be crucial to more accurately characterize environmental PFAS exposure and assess its potential health impacts on companion animals.

## 5 Conclusion

This study confirmed the presence of several PFAS compounds in canine and feline serum samples from Italian and Dutch cohorts. In dogs, higher PFOS concentrations were observed in animals from the Netherlands than in those from Italy, suggesting potential geographical differences in PFAS exposure patterns. However, no clear associations were identified between PFAS concentrations and the endocrine disorders investigated in the study populations. Future studies should therefore include larger sample sizes, well-defined healthy control groups, ideally from minimally contaminated areas, and more detailed exposure assessments.

The association between PFAS concentrations and age remains difficult to interpret and requires further investigation to better understand exposure dynamics and potential species-specific differences in PFAS distribution and elimination. Longitudinal studies across different life stages, together with comparisons with available human data while taking interspecies metabolic differences into consideration, may provide additional insights into PFAS bioaccumulation and biological effects.

## Data Availability

The raw data supporting the conclusions of this article will be made available by the authors, without undue reservation.
