# Supplementary material for: An investigation of per- and polyfluoroalkyl concentrations in dogs and cats with endocrine disorders
Source: Front Vet Sci. 2026 Aug 24;13:1904721. doi: 10.3389/fvets.2026.1904721 (PMC13548984; doi:10.3389/fvets.2026.1904721)
Supplement: Supplementary file 1 [file Table_1.docx]

Supplementary Material

**Table S1.** Chemical species belonging to the PFAS class analysed in the present study and their corresponding internal standards.

| Full name (abbreviation) | Formula | Molecular weight (g/mol) |
| --- | --- | --- |
| Perfluorobutanoic acid (PFBA) | C_4_HF_7_O_2_ | 213.97 |
| Perfluorobutanesulfonic acid (PFBS) | C_4_HF_9_O_3_S | 299.95 |
| Perfluoropentanoic acid (PFPeA) | C_5_HF_9_O_2_ | 263.98 |
| Perfluorohexanoic acid (PFHxA) | C_6_HF_11_O_2_ | 313.98 |
| Perfluoroheptanoic acid (PFHpA) | C_7_HF_13_O_2_ | 363.98 |
| Perfluorohexane sulfonic acid (PFHxS) | C₆F₁₃SO₃H | 400.11 |
| Perfluorooctanoic acid (PFOA) | C_8_HF_15_O_2_ | 413.97 |
| Perfluorooctane sulfonic acid (PFOS) | C_8_HF_17_O_3_S | 500.13 |
| Perfluorononanoic acid (PFNA) | C_9_HF_17_O | 463.97 |
| Perfluoro-n-decanoic acid (PFDA) | C_10_HF_19_O_2_ | 513.97 |
| Ammonium perfluoro-2-methyl-3-oxahexanoate (GenX) | C_6_H_4_F_11_NO_3_ | 329.98 |
| Perfluorooctanoic acid ^13^C_2_(^13^C_2_-PFOA) | ¹³C_2_C_6_HF_15_O_2_ | 416.97 |
| Perfluorooctane sulfonic acid ^13^C_4_ (^13^C_4_-PFOS) | ¹³C_4_C_4_HF_17_O_3_S | 504.00 |
| Ammonium perfluoro-2-methyl-3-oxahexanoate ^13^C_3_ (^13^C_3_-GenX) | ¹³C_3_C_3_H_4_F_11_NO_3_ | 333.05 |
