# Supplementary material for: An investigation of per- and polyfluoroalkyl concentrations in dogs and cats with endocrine disorders
Source: Front Vet Sci. 2026 Aug 24;13:1904721. doi: 10.3389/fvets.2026.1904721 (PMC13548984; doi:10.3389/fvets.2026.1904721)
Supplement: Supplementary file 2 [file Table_2.docx]

Supplementary Material

**Table S2.** Descriptive statistics of the canine study population of Bologna. Continues variables are reported as median (range), whereas categorical variables are presented as counts (n). ND=no data available.

| Age | 10 years (2-18) |  |  |
| --- | --- | --- | --- |
| BCS | 5/9 (3/9-8/9) |  |  |
| Gender | 69 females (56 spayed) | 57 males (25 castrated) |  |
| Breed | 42 mixed breed | 84 purebred |  |
| Housing status | 41 indoor | 19 outdoor | 66 ND |
| Type of diet | 80 commercial | 29 home-prepared | 17 ND |
| Comorbidities | 81 present | 45 absent |  |
| Tumors | 41 present | 28 absent | 57 ND |
