# Supplementary material for: An investigation of per- and polyfluoroalkyl concentrations in dogs and cats with endocrine disorders
Source: Front Vet Sci. 2026 Aug 24;13:1904721. doi: 10.3389/fvets.2026.1904721 (PMC13548984; doi:10.3389/fvets.2026.1904721)
Supplement: Supplementary file 3 [file Table_3.docx]

Supplementary Material

**Table S3.** Descriptive statistics of the feline population. Continues variables are reported as median (range), whereas categorical variables are presented as counts (n). ND=no data available.

| Age | 14 years (8-22) |  |  |
| --- | --- | --- | --- |
| BCS | 4/9 (1/9-8/9) |  |  |
| Gender | 42 females (39 spayed) | 42 males (38 castrated) |  |
| Breed | 75 domestic shorthair | 6 purebred |  |
| Housing status | 56 indoor | 20 outdoor | 8 ND |
| Type of diet | 69 commercial | 4 home-prepared | 11 ND |
| Comorbidities | 70 present | 14 absent |  |
| Tumors | 24 present | 17 absent | 43 ND |
