# Supplementary material for: An investigation of per- and polyfluoroalkyl concentrations in dogs and cats with endocrine disorders
Source: Front Vet Sci. 2026 Aug 24;13:1904721. doi: 10.3389/fvets.2026.1904721 (PMC13548984; doi:10.3389/fvets.2026.1904721)
Supplement: Supplementary file 4 [file Table_4.docx]

Supplementary Material

**Table S4.** Comparison of PFAS concentrations (µg/L) between the canine population of Bologna and Utrecht. Results are shown as median and range.

| Compounds | Population from Bologna (µg/L) | Population from Utrecht (µg/L) |
| --- | --- | --- |
| PFOS | 1.458 (0.050-23.465) | 2.511 (0.140-26.596) |
| PFOA | 0.139 (0.000-8.050) | 0.317 (0.000-2.757) |
| PFDA | 0.131 (0.000-8.046) | 0.151 (0.042-1.108) |
| PFNA | 0.150 (0.000-3.098) | 0.050 (0.000-2.692) |
| PFBS | 0.050 (0.000-1.211) | 0.050 (0.000-1.183) |
| PFHxS | 0.338 (0.000-3.781) | 0.655 (0.000-4.873) |
| PFHxA | 0.000 (0.000-0.000) | 0.000 (0.000-0.000) |
| PFHpA | 0.000 (0.000-0.102) | 0.000 (0.000-0.000) |
| PFPeA | 0.000 (0.000-0.050) | 0.000 (0.000-0.050) |
| GenX | 0.000 (0.000-0.000) | 0.000 (0.000-0.584) |
| PFBA | 0.000 (0.000-0.050) | 0.000 (0.000-0.050) |
